# Associations of Residential Greenness with Diabetes Mellitus in Chinese Uyghur Adults

**DOI:** 10.3390/ijerph16245131

**Published:** 2019-12-16

**Authors:** Shujun Fan, Zhenxiang Xue, Jun Yuan, Ziyan Zhou, Yuzhong Wang, Zhicong Yang, Boyi Yang, Guanghui Dong, Zhoubin Zhang

**Affiliations:** 1 Guangzhou Center for Disease Control and Prevention, Guangzhou 510440, China; fanfan0721ykl@163.com (S.F.); yuanjuncdc@163.com (J.Y.); zhouziyan1210@163.com (Z.Z.); yangzc@gzcdc.org.cn (Z.Y.); 2 Shufu Center for Disease Control and Prevention, Kashgar 844100, China; shfxmygh@163.com (Z.X.); yuzhongwangcdc@163.com (Y.W.); 3 Guangzhou Key Laboratory of Environmental Pollution and Health Risk Assessment, Guangdong Provincial Engineering Technology Research Center of Environmental and Health Risk Assessment, Department of Occupational and Environmental Health, School of Public Health, Sun Yat-sen University, Guangzhou 510080, China; yangby23@mail.sysu.edu.cn

**Keywords:** greenness, diabetes, fasting glucose, Uyghur

## Abstract

Greenness exposure is nominated as a potential beneficial factor for health, but evidence is limited on its diabetes effects. We conducted a cross-sectional study between May and September 2016 in rural areas of northwestern China, including 4670 Uyghur adults, to explore the associations between residential greenness and fasting glucose levels and diabetes prevalence. Fasting glucose levels were determined, and information on covariates was collected by questionnaire. Normalized difference vegetation index (NDVI) and soil-adjusted vegetation index (SAVI) were calculated to assess greenness levels. Generalized linear mixed models were applied to evaluate the associations of greenness with fasting glucose levels and diabetes prevalence. The prevalence of diabetes was 11.6%. We found that living in rural areas characterized by increased amounts of greenness was associated with reduced diabetes prevalence (e.g., NDVI_1000m_: OR, 0.92; 95% CI, 0.86, 0.99). Stratified analyses showed that the protective effects of greenness on diabetes prevalence were found only in women (NDVI_1000m_: OR, 0.90; 95% CI, 0.82, 0.99). However, none of the interaction was statistically significant. Our study suggests that greater residential greenness levels were associated with a lower odds ratio of diabetes prevalence in Xinjiang Uyghur adults. Further well-designed longitudinal studies are needed to confirm our findings.

## 1. Introduction

Diabetes mellitus is one of the major chronic diseases worldwide, and its prevalence has increased in recent years [1]. In China, diabetes mellitus has also become a public health problem, and the most recent national representative survey showed that the overall prevalence of diabetes in Chinese adults has increased from 9.7% in 2007 to 11.6% in 2010, representing an estimated 114 million Chinese adults with diabetes mellitus [2]. Therefore, there is an urgent need to take effective intervention strategies to control the uprising trend.

The etiology of diabetes mellitus is complex and is thought to be influenced by both genetic and environmental factors [3]. For environmental factors, high-sugar, high-fat, and high-energy diet habits, lack of physical activity, being overweight/obese, and exposure to air pollution have been reported to increased risk of diabetes mellitus [4,5,6]. Recently, diabetic effects of greenness have attracted more and more researchers’ attention worldwide. Mechanistically, greenness can bring health benefits by reducing levels of ambient air pollutants, extreme heat waves, and noise exposure, by encouraging physical activity, by reducing adiposity, and by enriching microbial diversity [7]. Several studies have explored the associations of greenness exposure with glucose levels, insulin resistance, and diabetes mellitus risk, but showed inconsistent results [8,9,10,11,12,13,14,15,16,17,18,19,20]. For example, cross-sectional studies conducted in Netherlands, Australia, the United States, the United Kingdom, and Canada have found that green space had a protective effect on diabetes mellitus [13,14,15,17,18,20], while a cross-sectional study among the Norwegian population found no significant association [8]. Two prospective cohort studies from England and Canada observed a significant association between increased greenness exposure and lower relative hazard of developing diabetes mellitus [9,16], but the Framingham study did not find such a longitudinal association [11]. While most prior studies were conducted among adults, a few have also explored potential diabetic effects of greenness among children and adolescents [12,19]. For example, a study of 837 German adolescents identified significant associations between higher greenness and lower insulin resistance [12]. Similarly, in a study of 3844 Iranian children, Dadvand et al. observed inverse associations between time spent in green spaces and fasting blood glucose levels and the risk of impaired fasting glucose [19]. Despite this, the prior studies were mostly conducted in developed countries, and only one study was conducted in China [10].

Additionally, although most prior studies on greenness and health were conducted in urban areas, few were performed among people living in rural areas (i.e., unurbanized areas like countryside or village). Xinjiang Uyghur Autonomous Region is located in northwest of China and is the largest province in China. The Uyghur is the largest inhabitant minority group in the province, whose lifestyle, culture, religion, genetic, and diet habits are different from Han ethnicities. It is also usually reported that the prevalence of diabetes mellitus is high in this region [21]. Further, Xinjiang belongs to the typical temperate continental arid climate, where it is dry, receiving little rainfall (with the mean annual precipitation of 100 mm in Southern Xinjiang), with little green space [22]. Additionally, rural areas in Xinjiang are economically backward with low health service, and people may have low health awareness, thus, rural population may be more vulnerable to environmental exposure [23]. Therefore, the aim of the current study was to evaluate the effects of residential greenness on diabetes prevalence and fasting glucose levels among rural Uyghur adults in Xinjiang Uyghur Autonomous Region.

## 2. Materials and Methods

### 2.1. Study Participants and Inclusion Criteria

The current study was conducted between May and September 2016 in Kashgar city. The city is an important economic, political, and cultural center in northwestern China. Due to Xinjiang having vast land area, low population density, and inconvenient transportation, we adopted a multistage city–county–towns–village stratified cluster random sampling method to select participants for saving cost. We chose Shufu county of Kashagar city as the study area. Shufu county consists of 10 towns with 123 villages (16 villages in Bulacksou town, 11 in LanGan town, 9 in Mushi town, 16 in Sayibarg town, 16 in Tashmeric town, 4 in Tierimu town, 8 in Toquzak town, 16 in WuPal town, 8 in Wucusack town, and 19 in Zhanmin town). Population ranged between 2000 and 30,000 among the villages. Over 290,000 people reside in Shufu county, of whom 98% are Uyghurs. First, we selected one village from each of the 10 towns using the random number method. Second, from each study village, all adults aged ≥18 years old and who had lived in the village for at least two years were selected for study enrollment.

A total of 5087 potential individuals were invited to take part in the current study, of whom 4772 individuals returned the questionnaire, yielding a response rate of 93.81%. We excluded 102 individuals due to having malignant diseases (e.g., chronic liver disease, renal failure, or cancer), being pregnant, and absence of a blood sample for glucose testing. Finally, a total of 4670 participants were included in the current analysis. The study procedures and protocols were reviewed and approved by the Ethics Committee of Guangzhou Center for Disease Control and Prevention prior to the present study (Identification code: GZCDC-ER[A]2016007). Written informed consent was obtained from all participants before all survey data and specimens were collected.

### 2.2. Data Collection and Diabetes Mellitus Definition

We used a self-administered questionnaire delivered by a face-to-face interview to collect information on demographic characteristics (e.g., age, sex, education levels, and marital status), tobacco use and alcohol drinking status, and physical activity levels. As detailed in our previous paper [24], tobacco use and alcohol drinking status were categorized as current, former, and non-smokers or drinkers, while physical activity levels were categorized as low, moderate, and high levels.

All participants were asked for permission to collect a blood sample for biochemical analysis after an overnight fasting (≥8 h). Fasting glucose concentration was determined by an enzymatic colorimetric method, using a Roche Autoanalyzer (Cobas c702 type; Roche Ltd.; Mannheim, Germany) in Guangzhou Center for Disease Control and Prevention.

Diabetes mellitus is defined as the fasting glucose level ≥7.0 mmol/L and/or a physician diagnosis and/or currently receiving pharmacological treatment for diabetes, according to the recommendations of the American Diabetes Association [25].

### 2.3. Confounders and Mediators

We chose confounders according to the recommendations by Jager et al. [26], in which a confounder has to meet the following three criteria: (1) it should be a risk factor for diabetes or elevated blood glucose; (2) it must be a cause of the greenness exposure and unequally distributed among participants with different greenness levels; and (3) it must not be an “effect” of greenness exposure, nor be an intermediate factor in the causal pathway of diabetes. Then, we developed a directed acyclic graph (DAG, Figure S1) to select a minimally sufficient set of covariates to adjust for confounding [27]. According to the DAG, the following confounders were retained: age, sex, marital status, and educational levels. Also, physical activity was chosen as a potential mediator (Figure S1).

### 2.4. Residential Greenness

We defined residential greenness using two satellite-based vegetation indexes, normalized difference vegetation index (NDVI) [28] and soil-adjusted vegetation index (SAVI) [29]. Derivations of both NDVI and SAVI indexes were calculated according to the land surface reflectance of the visible red and near-infrared parts of the light wavelengths. SAVI is similar to NDVI while additionally includes a correction factor to minimize soil influences. Both NDVI and SAVI values range from −1 (water) to +1 (completely vegetated areas). We used two cloud-free Landsat 5 Thematic Mapper satellite images at a resolution of 30 m (http://earthexplorer.usgs.gov) obtained in September 2017 to calculate the greenness levels. Both NDVI and SAVI were defined as mean values in circular buffers of 100 m, 300 m, 500 m, and 1000 m around each participant’ residential address centroid. These calculations were conducted by the ArcGIS 10.4 (ESRI, Redlands, CA, USA).

### 2.5. Statistical Analysis

Means and standard deviations were used to describe normally distributed continuous variables (e.g., age, body mass index (BMI), and fasting glucose levels). Frequency and percentage were used to describe the categorical variables (e.g., sex, education level, marital status, smoking and drinking status, and physical activity). Student’s *t*-tests were used to test differences in mean levels for continuous normally distributed variables between non-diabetes mellitus and diabetes mellitus groups, and chi-square tests were used to test differences in percentages for categorical variables. Spearman correlation analysis was performed to evaluate the intercorrelations between NDVI and SAVI.

Generalized linear mixed models (GLMMs) were used to explore the association between greenness with fasting glucose levels and diabetes mellitus prevalence, as described previously [10]. In the models, study village was incorporated as random effect and greenness and covariates were included as fixed effects. The effect estimates are presented as regression coefficients (β) and odds ratios (OR) and their corresponding 95% confidence intervals (CI). Basic regression models were unadjusted, and we further evaluated models with adjustment for confounders selected using DAG. We also built models with additional adjustment for smoking and alcohol status to explore their potential impact. Then, we categorized NDVI_1000m_ and SAVI_1000m_ into quartiles to test for nonlinear relationships. Additionally, we assessed the associations between greenness and fasting glucose levels with diabetes mellitus by sex (female vs. male) and age (<48 years vs. ≥48 years). We also explored potential mediation effects of physical activity by comparing the effect estimates before and after additional adjustment for physical activity [30]. For each model, effect estimates for fasting glucose levels and diabetes mellitus were calculated per interquartile range (IQR) increase in NDVI and SAVI. All statistic analyses were performed on SAS software (version 9.4; SAS institute, Inc. Cary, NC, USA). Levels of statistical significance were set at an α level of 0.05.

## 3. Results

### 3.1. Study Population Characteristics

Mean age of the participants was 47.2 years old, and 64.7% were women (Table 1). About half of the participants (49.3%) had low educational level (i.e., primary school), 88.6% were self-reported nonsmokers, 97.0% were self-reported nondrinkers, and 93.2% had low physical activity level. The overall prevalence of diabetes mellitus was 11.6%, of whom 110 reported a physician-diagnosed diabetes or were receiving antidiabetic medicine. Compared with individuals without diabetes mellitus, those with diabetes mellitus manifested significantly higher age, BMI, and fasting glucose levels, and were more likely to be male and have lower education levels (no school and primary school) (all *p* < 0.05).

Table 2 shows distributions and correlations of NDVI and SAVI. Greenness levels of the study participants differed markedly among the study participants. For example, NDVI_1000m_ ranged from 0.28 to 0.57 with a median value of 0.47. Additionally, we observed that NDVI was significantly correlated with SAVI, and Spearman’s correlation coefficients ranged from 0.40 to 0.99 (all *p* < 0.01).

### 3.2. Relationship of Greenness with Fasting Glucose and Diabetes Mellitus

Table S1 and Table 3 summarize the results of linear regression analysis for greenness with fasting glucose levels in crude and adjusted models, respectively. We observed that there was no significant association for any buffers of NDVI and SAVI with fasting glucose levels.

In crude logistic regression analysis, an IQR increase in NDVI_1000m_ and SAVI_1000m_ was significantly associated with 7% (NDVI_1000m_: OR = 0.93, 95% CI = 0.87, 1.00; SAVI_1000m_: OR = 0.93, 95% CI = 0.87, 0.99) reduction in odds for diabetes risk (Table S1). The effect estimates were not influenced by further adjustments for age, sex, marital status, and education levels (Table 3). For example, an IQR increase in NDVI_1000m_ and SAVI_1000m_ was associated with 8% (both OR = 0.92, 95% CI = 0.86, 0.99) reduction in odds for diabetes risk. However, no significant associations were found for NDVI and SAVI in smaller buffers.

We additionally adjusted the main models for smoking and alcohol drinking status and found that the effect estimates were not materially changed (Table S2). Also, we performed mediation analysis but did not find any mediating role of physical activity (Table S3). Further, we categorized NDVI_1000m_ and SAVI_1000m_ into quartiles and found that effects estimates were lower in higher quartiles than in lower quartiles, and *p* values for trend were significant for all (Table S4).

### 3.3. Relationship of Greenness with Fasting Glucose and Diabetes Mellitus Stratified by Sex and Age

Table 4 shows the sex- and age-stratified associations for residential greenness and fasting glucose levels. We found that the relationship of residential greenness with fasting glucose levels failed to reach significance in both sex and age subgroups. And the *p* values for the interactions were not significant.

Table 5 shows the results of analyses for residential greenness in relation to diabetes mellitus stratified by sex and age. The protective association of greenness and diabetes prevalence was only found in women (e.g., IQR increases in NDVI_1000m_ and SAVI_1000m_ were both associated with a 10% (NDVI_1000m_: OR = 0.90, 95% CI = 0.82, 0.99; SAVI_1000m_: OR = 0.90, 95% CI = 0.82, 0.98) reduction in odds for diabetes mellitus prevalence). However, none of the interactions were statistically significant. When the analysis was stratified by age, the relationship of residential greenness with diabetes prevalence failed to reach significance in both <48 and ≥48 age groups.

## 4. Discussion

The findings from the population-based study of 4670 Uyghur adults showed that living in greener areas was associated with reduced diabetes mellitus prevalence. In addition, neither age nor sex significantly modified the associations. This is the first study to examine the relationship between residential greenness and diabetic metrics among Uyghur rural population.

The relationships of greenness and diabetes mellitus have received a great deal of attention during the past decade. For instance, Maas et al. conducted a large cross-sectional study of 345,143 individuals in Dutch and found that the annual prevalence rate of diabetes mellitus was lower in people living in areas with higher green space levels [17]. Another study of 267,072 adult Australians (≥45 years old) found that the rate of self-report diabetes mellitus was decreased with the increased green space of residential neighborhoods [13]. A study of 249,405 USA elderly (≥65 years) also found greenness significantly reduced diabetes mellitus risk; an increase in NDVI from 1 SD less to 1 SD more than mean was associated with a reduced risk of 14% for diabetes mellitus [14]. Likewise, another three cross-sectional studies conducted in USA [15], the United Kingdom [18], and Canada [20] observed increased neighborhood or street green space was significantly associated with lower individual diabetes mellitus prevalence. Additionally, several cohort studies have also documented a significant and inverse relationship between greenness levels and diabetes mellitus incidence [9,16]. However, there are also a few studies that found no association between them [8,11]. Our research group has also investigated the association of greenness with diabetes mellitus in Chinese Han adults and found that higher residential greenness was significantly associated with a lower prevalence of diabetes mellitus [10]. Our current findings were consistent with most of the prior studies that residential greenness was associated with diabetes mellitus prevalence.

However, we did not find an association of residential greenness with fasting glucose levels. To the best of our knowledge, there were only three studies that have explored association between them. Specifically, in a cross-sectional study of 3844 Iranian schoolchildren aged 7–18 years, Dadvand et al. found that more time spent in green spaces, especially in natural green spaces, was significantly associated with decreased fasting glucose levels [19]. In the Framingham Heart Study, Lee and colleagues showed that higher green space was associated with lower fasting glucose levels in cross-sectional analysis, although the association disappeared in longitudinal analysis [11]. Additionally, the cross-sectional study conducted in the Chinese adults found that a 0.1-unit increase in NDVI_500m_ was significantly associated with a 1.14% reduction in fasting glucose levels [10]. Inconsistent with these cross-sectional findings, our current study did not observe an inverse association of residential greenness with fasting glucose levels among Uyghur adults. Additionally, we did not detect a significant modification effect of sex and age on the associations between greenness and diabetes. The current evidence in this topic is mixed. For example, consistent with our findings, Dadvand et al. [19] did not find a difference in the strength of association of greenness and fasting glucose between Iranian boys and girls. However, in the study by Yang et al., which we have already described, stronger associations of greenness with fasting glucose levels were observed in men than in women [10]. Also, unlike our study, Yang et al. reported that the association of greenness with diabetes mellitus prevalence was stronger in younger participants, while no effect modification by age was observed for fasting glucose levels [10]. Further studies with large sample size and longitudinal design therefore are needed to validate these findings.

The precise mechanisms by which greenness benefits health are not fully understood. Several investigators have proposed that green spaces might have beneficial effects on glucose homeostasis markers and the development of diabetes mellitus via actively filtering out ambient air pollutions levels [12]. Ambient air pollutions, such as NO, NO_2_, PM_1_, PM_2.5_, and PM_10_, were significantly associated with increased risk of diabetes mellitus, which was observed in many previous studies [5,31,32]. Green space would reduce ambient air pollutions levels. Thus, the association of residential greenness with fasting glucose and diabetes mellitus risk may be plausible. Furthermore, previous studies reported a significant negative relationship of green space with adiposity [33,34], which is a major risk factor for diabetes mellitus. Therefore, it is also proposed that greenness may benefit glucose metabolism by reducing adiposity. Reducing physical activity is also proposed as a potential mechanism by which greenness affects health. We explored mediation analysis to explore this but failed to detect a mediation role of physical activity. Additionally, greenness was also associated with psychosocial stress, reduced noise exposure, and greater and more diverse microbial exposure, all of them might influence glucose homeostasis and the development of diabetes mellitus [9,35,36,37].

Our study has several limitations, which need to be acknowledged. First, the cross-sectional design of the study precludes us to establish a temporal association between greenness exposure with fasting glucose levels and diabetes mellitus risk. However, the possibility of the reverse causality, that is, people with diabetes moved to areas with lower greenness levels, is very low. Second, although the sample size of our study was relatively large, the statistical power remained not ideal and thus most associations were not statistically significant, especially in linear regression analyses. Therefore, future well-designed studies with large sample size are expected to validate our findings. Third, although we calculated greenness exposure levels using high-resolution satellite images, the NDVI and SAVI just represent general vegetation levels but cannot distinguish the type, utility, structure, and quality of green space, which also may lead to exposure misclassification. Fourth, data on covariates were collected by questionnaire, so recall bias cannot be ignored because the responses might be subject to the participant’s memory influence. Fifth, although we considered and adjusted for some confounders, information on potential mediators or confounders, such as air pollutions, mental stress, dietary habits, noise exposure, and time spent of green space, were unavailable in the study. Therefore, we cannot further explore potential mechanisms underlying the association between greenness and diabetes, and residual confounding caused by unmeasured confounders (e.g., village-level income) remains possible. Finally, we defined diabetes based on a single point measurement of fasting glucose, and detailed data on fasting period were not available, which might have caused outcome misclassification and precludes us from performing sensitivity analyses. Nonetheless, our study still has several apparent strengths. First, the participants are homogenous, which improves the effectiveness of statistical analysis, because they were of Uyghur nationality and from the same region of Kashgar. Second, to the best of our knowledge, our findings provide the first evidence on the association of residential greenness and diabetes mellitus risk in Uyghur population in Kashgar region.

## 5. Conclusions

In summary, exposure to higher residential greenness (large buffer in particular) was significantly associated with a lower odds ratio of diabetes mellitus in Xinjiang Uyghur nationality adults. However, further well-designed longitudinal studies with larger sample size are needed to confirm our findings when considering the limitations of our study.

## Figures and Tables

**Table 1:** Characteristics of the study participants.

Variables	No Diabetes Mellitus (*n* = 4127)	Diabetes Mellitus (*n* = 543)	*p*
Age, years	46.4 ± 14.2	53.6 ± 13.1	<0.001
Sex, *n* (%)			
Men	1418 (34.4)	229 (42.2)	<0.001
Women	2709 (65.6)	314 (57.8)	
Education level, *n* (%)			
No school	370 (9.0)	78 (14.4)	<0.001
Primary school	2002 (48.5)	299 (55.1)	
Middle school	1497 (36.3)	136 (25.1)	
>Junior college	258 (6.3)	30 (5.5)	
Marital status, *n* (%)			
Not married	164 (4.0)	9 (1.7)	0.025
Get married	3577 (86.7)	479 (88.2)	
Divorce or widowed	386 (9.4)	55 (10.1)	
Smoking status, *n* (%)			
Non-smokers	3662 (88.7)	474 (87.3)	0.443
Former-smokers	380 (9.2)	59 (10.9)	
Current-smokers	85 (2.1)	10 (1.8)	
Drinking status, *n* (%)			
Non-drinkers	3998 (96.9)	533 (98.2)	0.238
Former-drinkers	81 (2.0)	7 (1.3)	
Current-drinkers	48 (1.2)	3 (0.6)	
Physical activity, *n* (%)			
Low strength	3848 (93.2)	506 (93.2)	0.963
Moderate or high strength	279 (6.8)	37 (6.8)	
Body mass index, kg/m^2^	24.9 ± 4.7	26.3 ± 4.8	<0.001
Fasting glucose, mmol/L	5.3 ± 0.7	9.6 ± 4.5	<0.001

**Table 2:** Distributions and intercorrelations (Spearman correlation coefficients) for NDVI and SAVI.

	Median (IQR)	Min	Max	NDVI_100m_	NDVI_300m_	NDVI_500m_	NDVI_1000m_	SAVI_100m_	SAVI_300m_	SAVI_500m_	SAVI_1000m_
NDVI_100m_	0.45 (0.20)	0.20	0.67	1							
NDVI_300m_	0.48 (0.12)	0.21	0.63	0.74 **	1						
NDVI_500m_	0.47 (0.09)	0.23	0.57	0.57 **	0.88 **	1					
NDVI_1000m_	0.47 (0.06)	0.28	0.57	0.42 **	0.63 **	0.82 **	1				
SAVI_100m_	0.29 (0.14)	0.13	0.44	0.99 **	0.74 **	0.55 **	0.42 **	1			
SAVI_300m_	0.32 (0.08)	0.14	0.42	0.71 **	0.99 **	0.90 **	0.63 **	0.71 **	1		
SAVI_500m_	0.32 (0.06)	0.15	0.39	0.53 **	0.86 **	0.99 **	0.79 **	0.52 **	0.89 **	1	
SAVI_1000m_	0.32 (0.04)	0.19	0.39	0.41 **	0.64 **	0.84 **	0.99 **	0.40 **	0.65 **	0.82 **	1

Abbreviations: IQR, interquartile range (computed by subtracting the first quartile from the third quartile); Max, maximum; Min, minimum; NDVI, normalized difference vegetation index; SAVI, soil-adjusted vegetation index. ** Correlation is significant at the 0.01 level (two-tailed).

**Table 3:** Associations of NDVI and SAVI (per IQR increase) around the residential address with fasting glucose levels and diabetes mellitus prevalence (adjusted models).

	Fasting Glucose		Diabetes Mellitus	
	β (95% CI) *	*p*	OR (95% CI) *	*p*
NDVI_100m_	0.04 (−0.07, 0.14)	0.487	0.97 (0.83, 1.13)	0.683
NDVI_300m_	−0.01 (−0.08, 0.07)	0.864	0.92 (0.82, 1.03)	0.128
NDVI_500m_	−0.01 (−0.08, 0.05)	0.657	0.92 (0.84, 1.01)	0.089
NDVI_1000m_	−0.02 (−0.06, 0.03)	0.531	0.92 (0.86, 0.99)	0.024
SAVI_100m_	0.07 (−0.04, 0.18)	0.238	1.00 (0.85, 1.17)	0.966
SAVI_300m_	−0.01 (−0.09, 0.07)	0.836	0.92 (0.82, 1.03)	0.132
SAVI_500m_	−0.02 (−0.08, 0.04)	0.540	0.92 (0.84, 1.01)	0.075
SAVI_1000m_	−0.02 (−0.07, 0.03)	0.458	0.92 (0.86, 0.99)	0.020

Abbreviations: IQR, interquartile range; NDVI, normalized difference vegetation index; SAVI, soil-adjusted vegetation index; OR, odds ratio. * Adjusted for age, sex, marital status, and educational levels.

**Table 4:** Sex- and age-stratified associations between per IQR increase in NDVI and SAVI around the residential address with fasting glucose levels (adjusted models).

	Males	Females	<48	≥48
	β (95% CI) *	β (95% CI) *	β (95% CI) *	β (95% CI) *
NDVI_100m_	0.07 (−0.10, 0.24)	0.00 (−0.13, 0.13)	0.08 (−0.04, 0.19)	0.01 (−0.15, 0.16)
NDVI_300m_	−0.04 (−0.17, 0.09)	0.01 (−0.09, 0.10)	0.05 (−0.03, 0.14)	−0.04 (−0.15, 0.08)
NDVI_500m_	−0.06 (−0.16, 0.05)	0.01 (−0.07, 0.09)	0.02 (−0.06, 0.09)	−0.02 (−0.11, 0.07)
NDVI_1000m_	−0.04 (−0.12, 0.05)	−0.01 (−0.07, 0.05)	−0.03 (−0.08, 0.03)	0.00 (−0.07, 0.07)
SAVI_100m_	0.11 (−0.08, 0.29)	0.03 (−0.01, 0.17)	0.11 (−0.01, 0.23)	0.04 (−0.13, 0.20)
SAVI_300m_	−0.03 (−0.16, 0.10)	0.00 (−0.09, 0.10)	0.05 (−0.03, 0.14)	−0.04 (−0.15, 0.08)
SAVI_500m_	−0.06 (−0.16, 0.05)	−0.01 (−0.08, 0.08)	0.01 (−0.06, 0.09)	−0.03 (−0.12, 0.07)
SAVI_1000m_	−0.04 (−0.12, 0.04)	−0.01 (−0.07, 0.05)	−0.03 (−0.08, 0.03)	−0.01 (−0.08, 0.07)

Abbreviations: IQR, interquartile range; NDVI, normalized difference vegetation index; SAVI, soil-adjusted vegetation index. * Adjusted for age, marital status, and educational levels.

**Table 5:** Sex- and age-stratified associations between per IQR increase in NDVI and SAVI around the residential address with diabetes mellitus prevalence (adjusted models).

	Males	Females	<48	≥48
	OR (95% CI) *	OR (95% CI) *	OR (95% CI) *	OR (95% CI) *
NDVI_100m_	1.01 (0.79, 1.28)	0.94 (0.77, 1.15)	0.85 (0.61, 1.17)	1.01 (0.85, 1.20)
NDVI_300m_	0.96 (0.80, 1.14)	0.87 (0.76, 1.03)	0.93 (0.73, 1.19)	0.92 (0.81, 1.05)
NDVI_500m_	0.96 (0.83, 1.02)	0.90 (0.80, 1.01)	0.95 (0.77, 1.16)	0.93 (0.84, 1.03)
NDVI_1000m_	0.95 (0.85, 1.07)	0.90 (0.82, 0.99)	0.89 (0.76, 1.03)	0.94 (0.87, 1.02)
SAVI_100m_	1.05 (0.81, 1.35)	0.96 (0.77, 1.20)	0.88 (0.62, 1.24)	1.04 (0.86, 1.26)
SAVI_300m_	0.96 (0.80, 1.15)	0.89 (0.76, 1.03)	0.95 (0.74, 1.21)	0.92 (0.81, 1.05)
SAVI_500m_	0.95 (0.82, 1.11)	0.89 (0.79, 1.01)	0.95 (0.78, 1.17)	0.92 (0.83, 1.03)
SAVI_1000m_	0.95 (0.85, 1.07)	0.90 (0.82, 0.98)	0.90 (0.77, 1.05)	0.93 (0.86, 1.01)

Abbreviations: IQR, interquartile range; NDVI, normalized difference vegetation index; SAVI, soil-adjusted vegetation index, OR, odds ratio. * Adjusted for age, marital status, and educational levels.

## Supplementary Materials

The following are available online at https://www.mdpi.com/1660-4601/16/24/5131/s1, Figure S1: Directed acyclic graph for the association between greenness and diabetes. Pink lines indicate potential confounders, and green lines indicate potential mediators. Table S1: Associations of NDVI and SAVI (per IQR increase) around the residential address with fasting glucose levels and diabetes mellitus prevalence (crude models). Table S2: Associations of NDVI and SAVI (per IQR increase) around the residential address with fasting glucose levels and diabetes mellitus prevalence after additionally adjusting main models for smoking and alcohol drinking status. Table S3: Associations of NDVI and SAVI (per IQR increase) around the residential address with fasting glucose levels and diabetes mellitus prevalence after additionally adjusting main models for physical activity. Table S4: Associations of categorical NDVI and SAVI levels with fasting glucose levels and diabetes mellitus prevalence.
