# Supplementary material for: Associations of Residential Greenness with Diabetes Mellitus in Chinese Uyghur Adults
Source: Int J Environ Res Public Health. 2019 Dec 16;16(24):5131. doi: 10.3390/ijerph16245131 (PMC6950214; doi:10.3390/ijerph16245131)
Supplement: Supplementary file 1 [file ijerph-16-05131-s001.pdf]

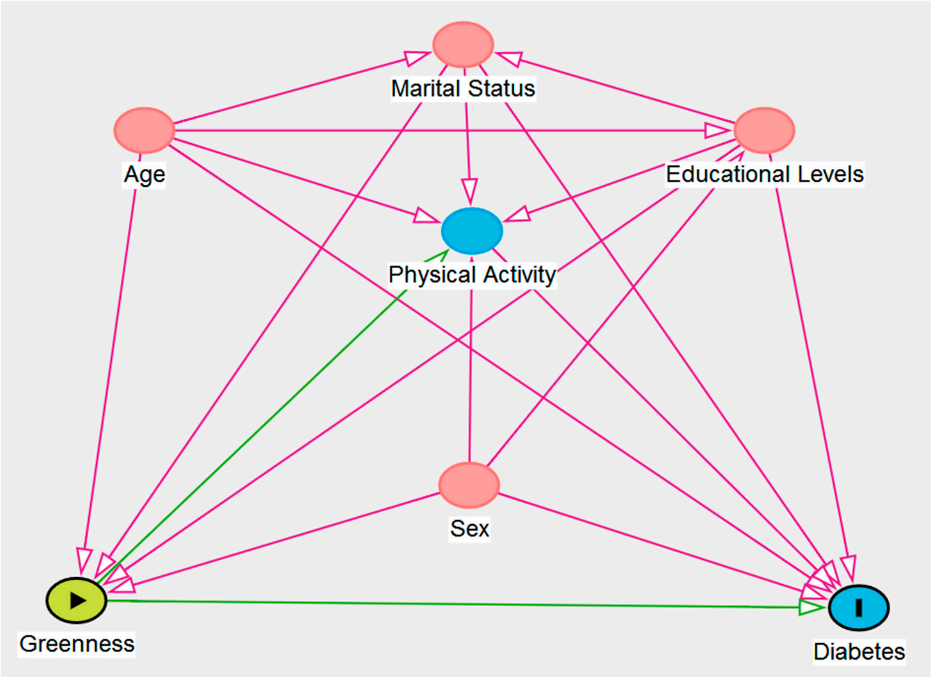

Figure S1. Directed acyclic graph for the association between greenness and diabetes. Pink lines indicate potential confounders and green lines indicate potential mediators.

Table S1. Associations of NDVI and SAVI (per IQR increase) around the residential address with fasting glucose levels and diabetes mellitus prevalence (crude models)

	**Fasting glucose**		**Diabetes mellitus**	
	**Crude β (95% CI)**	***p***	**Crude OR (95% CI)**	***p***
$NDVI_{100\ m}$	0.02 (-0.08, 0.13)	0.671	0.92 (0.82, 1.03)	0.133
$NDVI_{300\ m}$	-0.01 (-0.09, 0.07)	0.782	0.92 (0.82, 1.03)	0.133
$NDVI_{500\ m}$	-0.01 (-0.08, 0.06)	0.772	0.93 (0.85, 1.02)	0.135
$NDVI_{1000\ m}$	-0.01 (-0.06, 0.04)	0.673	0.93 (0.87, 1.00)	0.039
$SAVI_{100\ m}$	0.05 (-0.06, 0.16)	0.358	0.98 (0.84, 1.15)	0.812
$SAVI_{300\ m}$	-0.01 (-0.09, 0.07)	0.780	0.92 (0.83, 1.03)	0.143
$SAVI_{500\ m}$	-0.01 (-0.08, 0.05)	0.674	0.93 (0.85, 1.02)	0.123
$SAVI_{1000\ m}$	-0.01 (-0.06, 0.04)	0.636	0.93 (0.87, 0.99)	0.039

Abbreviation: IQR, interquartile range; NDVI, Normalized Difference Vegetation Index; SAVI, Soil Adjusted Vegetation Index; OR, odds ratio.

Table S2. Associations of NDVI and SAVI (per IQR increase) around the residential address with fasting glucose levels and diabetes mellitus prevalence after additionally adjusting main models for smoking and alcohol drinking status

	**Fasting glucose**		**Diabetes mellitus**	
	**β (95% CI)**	*p*	**OR (95% CI)**	*p*
$NDVI_{100\ m}$	0.04 (-0.07, 0.14)	0.491	0.97 (0.83, 1.13)	0.668
$NDVI_{300\ m}$	-0.06 (-0.08, 0.07)	0.880	0.92 (0.82, 1.03)	0.135
$NDVI_{500\ m}$	-0.01 (-0.08, 0.05)	0.677	0.92 (0.84, 1.01)	0.095
$NDVI_{1000\ m}$	-0.01 (-0.06, 0.03)	0.288	0.92 (0.86, 0.99)	0.027
$SAVI_{100\ m}$	0.06 (-0.04, 0.18)	0.241	1.00 (0.84, 1.17)	0.951
$SAVI_{300\ m}$	-0.01 (-0.08, 0.07)	0.855	0.92 (0.82, 1.03)	0.138
$SAVI_{500\ m}$	-0.02 (-0.08, 0.04)	0.557	0.92 (0.84, 1.01)	0.081
$SAVI_{1000\ m}$	-0.02 (-0.07, 0.03)	0.482	0.92 (0.86, 0.99)	0.023

Abbreviation: IQR, interquartile range; NDVI, Normalized Difference Vegetation Index; SAVI, Soil Adjusted Vegetation Index; OR, odds ratio.

Adjusted for age, sex, marital status, educational levels, and smoking and alcohol drinking status.

Table S3. Associations of NDVI and SAVI (per IQR increase) around the residential address with fasting glucose levels and diabetes mellitus prevalence after additionally adjusting main models for physical activity

	**Fasting glucose**		**Diabetes mellitus**	
	**β (95% CI)**	*p*	**OR (95% CI)**	*p*
$NDVI_{100\ m}$	0.03 (-0.07, 0.13)	0.571	0.97 (0.83, 1.13)	0.690
$NDVI_{300\ m}$	-0.02 (-0.10, 0.06)	0.606	0.91 (0.81, 1.03)	0.125
$NDVI_{500\ m}$	-0.03 (-0.10, 0.04)	0.393	0.92 (0.84, 1.01)	0.084
$NDVI_{1000\ m}$	-0.03 (-0.08, 0.03)	0.322	0.92 (0.85, 0.99)	0.022
$SAVI_{100\ m}$	0.06 (-0.05, 0.17)	0.300	1.00 (0.85, 1.18)	0.977
$SAVI_{300\ m}$	-0.02 (-0.10, 0.06)	0.574	0.91 (0.81, 1.03)	0.129
$SAVI_{500\ m}$	-0.03 (-0.10, 0.03)	0.301	0.92 (0.83, 1.01)	0.070
$SAVI_{1000\ m}$	-0.03 (-0.08, 0.02)	0.265	0.92 (0.85, 0.99)	0.019

Abbreviation: IQR, interquartile range; NDVI, Normalized Difference Vegetation Index; SAVI, Soil Adjusted Vegetation Index; OR, odds ratio.

Adjusted for age, sex, marital status, educational levels, and physical activity.

Table S4. Associations of categorical NDVI and SAVI levels with fasting glucose levels and diabetes mellitus prevalence

	**Fasting glucose**		**Diabetes mellitus**	
	**β (95% CI)**	*p for trend*	**OR (95% CI)**	*p for trend*
$NDVI_{1000\ m}$		0.013		0.002
Q1	Ref.		Ref.	
Q2	0.11 (-0.07, 0.30)		1.15 (0.89-1.48)	
Q3	-0.00 (-0.17, 0.17)		0.75 (0.58, 0.97)	
Q4	-0.11 (-0.29, 0.07)		0.75 (0.57, 0.99)	
$SAVI_{1000\ m}$		0.028		0.030
Q1	Ref.		Ref.	
Q2	0.06 (-0.11, 0.23)		0.96 (0.75, 1.23)	
Q3	-0.01 (-0.19, 0.17)		0.85 (0.65, 1.10)	
Q4	-0.07 (-0.25, 0.12)		0.76 (0.58, 1.00)	

Abbreviation: NDVI, Normalized Difference Vegetation Index; SAVI, Soil Adjusted Vegetation Index; OR, odds ratio.

Adjusted for age, sex, marital status, and educational levels.
